
JOURNAL INFORMATION
==============================
NLM Title Abbreviation: Med Chir Rev
NLM Journal ID: 0034617

The Medico-Chirurgical Review

Publisher: Biomedical Journal Digitization Project

ARTICLE INFORMATION
==============================
PMCID: PMC4966174
PMID: 29917597
Article version: 1
Subjects: Reviews

On Lunatic Asylums (Eclectic)

Print publication date: 1828 Jul 1
Volume: 9
Issue: 17
First page: 25
Last page: 34
1828 Apr 26
License: This work is free of known copyright restrictions. For more information, please see PMC Back Issue Digitization.
License URL: https://creativecommons.org/publicdomain/mark/1.0/

==============================

### III.

1. *General View of Lunatic Asylums, &c.* By Sir ANDREW HALLIDAY, M.D., &c.
2. *Practical Observations on Insanity, &c.* By W. J., Late a Keeper at a Lunatic Asylum.
3. *Outlines of Lectures on Mental Diseases.* By ALEXANDER MORISON, M.D.

The subject of Insanity and of Lunatic Asylums has lately engrossed so much attention, both in and out of Parliament,—both in and out of the profession, that we need offer no apology for dedicating an article to it in this place. That state in which the mind of man is in abeyance, and all his godlike faculties levelled in the dust—a state in which he is no longer able to protect or defend himself, imperiously claims our sympathy, and demands our most attentive consideration. "To behold the good, the great, the virtuous, and the brave, reduced by the withering influence of this awful visitation, to a level with the beasts of the field, on which the bright beams of reason never dawned, is, indeed, calculated to excite, in every feeling and philanthropic mind, sensations of the most painful nature." These painful emotions will not be mitigated, when we reflect that such unfortunate beings (still our fellow-creatures) have, for ages past, and even in this boasted land of liberty, been consigned to chains and dungeons, subjected to the care of mercenary wretches, who would scarcely have been tolerated to look after the felon, the murderer, or the most depraved and worthless portions of a redundant population!—All this, too, while pseudo-philanthropists have traversed the utmost limits of the empire, to inquire into the situation, and improve the condition of convicts—passing heedlessly by the cold and loathsome dungeon, where the guiltless and unhappy maniac was pining in hopeless captivity.

"It is true that a spirit of enquiry has been abroad of late; parliamentary committees have been instituted; the most atrocious cruelties have been brought to light; and it has been proved beyond the possibility of a doubt, that so far from any curative measures having been resorted to in the treatment of the insane, the method pursued has been such as must have had the effect of driving to irretrievable madness thousands who, under a system of ordinary mildness, would have been restored to society."

We are informed by Sir A. Halliday, who appears to have paid great attention to this subject for 25 years past, that it was not till 1774 that the attention of Parliament was drawn to Lunatics and Lunatic Asylums. In that year, an Act was passed, the inadequacy of which has been daily and hourly complained of ever since. It provides for the casual inspection of licensed establishments, but neither authorises the correction of abuses, nor permits the active interference of the visiting Commissioners, even where the most flagrant instances of mal-treatment are detected! That such an Act should have remained so long a blot on the Statute-book, might hereafter excite astonishment, were it a solitary blot—but, alas! the ignorance of law-makers is only equalled by the negligence of those to whom the execution of those laws is entrusted!

The little good that was done by this Act, to the wealthy inmates of licensed Asylums, was soon lost sight of—and it was never meant to apply to that helpless class of sufferers—*the insane poor of the kingdom.* These remained in a wretched and deplorable condition, when Sir Andrew, in a letter signed "MEDICUS," (1806) and addressed to Lord Henry Petty, induced Mr. Wynn to move for a Select Committee to investigate the subject. The result was an Act, authorising Magistrates to erect public asylums for the insane poor. After a lapse of seven years, a renewed inquiry took place, at the suggestion of Mr. Rose, and the evidence elicited upon this occasion, must be fresh in the memory of all. A Bill calculated to remedy the evil was three or four times passed through the Lower House—but rejected in the HOUSE of LORDS!! After Mr. Rose's death, it got into Chancery—and there it has slumbered for *nine years*—Lord Eldon not being able to make up his mind on the subject! Again the House of Commons has taken up the inquiry—and sincerely do we hope that justice, though tardy, may at length be done.

By Mr. Wynn's Act, the Magistrates, as we before observed, were empowered to erect, at the public expense, Asylums, or Hospitals for the treatment of the insane poor, under their own immediate inspection and government—but although twenty years have elapsed, it is only in the counties of York, Lancaster, Nottingham, Norfolk, Stafford, Bedford, Gloucester, Lincoln, Pembroke, and Cornwall—ten out of fifty-two counties, that asylums have been opened. The Magistrates of Middlesex, after two years' deliberation, have just announced that such an institution was necessary in a metropolitan county, where 873 of their fellow-creatures were proved to be suffering all the miseries of neglect and cruel treatment, from the want of such an institution!

"By parliamentary returns we learn that, in 1826 there were 1321 individuals in private asylums, exclusive of those in London and Westminster, and within seven miles thereof. And 1147 in public asylums, exclusive of those in St. Luke's and Bedlam, and 53 in public goals; giving a total for the several counties of England and Wales, of 2521. The private asylums, in and near the metropolis, may be taken at an average of 1750; they were, last year, 1761, and St. Luke's and Bedlam at 500: we have thus, for the whole of England, a total of 4782 insane persons, known to be in existence, and publicly accounted for according to law. But there is a number, if not equally great, at least nearly so, of whom the law takes no cognizance, and whose existence is only known to their relatives and friends. These consist of individuals placed in solitary confinement, with persons who take only one patient. This is a state of things that ought not to be allowed to remain as it is, for a single hour, in this land of boasted liberty. I do not say that it ever has taken place, though I have known one or two instances that might almost bear such a construction; but I maintain that it may take place, for there is no law to prevent it; that individuals have been sent into such seclusion, who never suffered from the pangs of madness, and it must be evident to every one who gives this subject the least consideration, that it only requires a faithful keeper and strict watchfulness to retain such persons in prison for life."

From a long and laborious investigation, Sir Andrew is convinced that the aggregate number of persons actually in confinement, in public and private asylums, and with their relations, or with individual keepers, in England and Wales, exceeds *eight thousand*—more than two thirds of whom are curable, and who, by proper means, might be restored to the enjoyments of social life. Sir A. does not think the malady is on the increase among us, as some authors have supposed. It is curious that insanity is most prevalent in the counties of York and Lancaster—in Wilts, Stafford, Durham, and Gloucester. Wales, in proportion to its population, has very few lunatics—"and the same remark holds good in respect to the *Celtic* tribes in other portions of the empire." The same author remarks that, where the said Celtic tribes have been little mixed with the Saxon or Norman blood, "insanity is scarcely known," except as arising from diseased structure or mal-formation of the cranium :—" idiots are therefore more frequent than lunatics." We have some doubts of the truth of the following position of our ingenious and learned author.

"It is not by seclusion and mystery, that they can be properly watched over, or by confinement in the wards or cells of an hospital, that they are to be cured. Experience has shewn that a *regulated intercourse with the world,* and constant employment of the mind and body, are the best aids to medical treatment, and in the construction of every asylum, these ought constantly to be kept in view."

We are no advocates for mystery, in any case ; but we can hardly think that "an intercourse with the world," is prudent, till the insanity is nearly, or very considerably abated. Whoever has gone into a lunatic asylum must have observed the instantaneous excitement called forth by the appearance of a stranger (and it would be far worse with a friend) amongst the unfortunate inmates of these dreary abodes. Every tongue is at work in pouring forth the injustice which is done them,—every nerve is strained in the attempt to convince the visiter of the reality of the ideal hallucination which has taken possession of the mind. Can these excitements and perpetual reiterations of the monomaniac impressions be conducive to a cure? We should think they are not. Is not the first and fundamental principle of treatment, in insanity, a removal from *friends*—and consequently from all sources of irritation? If an intercourse with *strangers* produces some degree of excitement or irritation, should not this intercourse be prohibited?—We think the following passage, from the same author, justifies this proposition.

"The best regulated public asylums in England, are those for the West Riding of York, at Wakefield, and for Lancaster. Bedlam, the great national or metropolitan asylum, is now well conducted, and the patients are humanely and judiciously treated ; but it has still too much of the leaven of the dark ages in its constitution, and too rigid a system of quackery is maintained, in regard to its being seen and visited by respectable strangers, and there is too little space for exercise and employment, for it ever to prove an efficient hospital. In some respects it is little better than when in fact, it formed one of the *lions* of the metropolis, and the patients, as wild beasts, were shewn at sixpence for each person admitted. I dislike, and decidedly condemn the practice of being carried round by a governor, and then asked to record an opinion of what we have seen and heard during our visit. It is the assumption of secresy that creates extraordinary curiosity, and probably, for a time, improper visiters might be drawn to the scene; but let Bedlam be as open to the public as the other hospitals in the kingdom, and none but those called by business or affection will ever think of asking for admission. St. Luke's, the next in importance to Bedlam, is only fit to become a prison for confirmed idiots. It is worse than useless as an hospital for curable lunatics :—not so much, I would observe, on account of what may be called the close borough system upon which it is managed (though even that merits condemnation in an establishment founded and endowed by a liberal public), as from its possessing none of the advantages now found most necessary for the recovery of the insane ;—and if ever St. Luke's Hospital is to be made available for the purposes for which its funds have been accumulated, it must be by adopting a plan similar to that which exists at Antwerp, and of which I shall give some account when I come to treat of the hospitals in the Netherlands."

Sir Andrew has thrown out many hints which may prove useful to those concerned in the erection of these asylums; and as medical men will, of course, be consulted by the magistrates on these occasions, we would recommend them to peruse our author's work in the original. We shall glance at a few of our author's observations.

He remarks that the great objection to the majority of asylums in England, is the want of space for work-shops—and of ground for agricultural labour. At Wakefield, the patients have uniformly been kept employed at their various trades, and in rural occupations, with the best effects. Dr. Ellis informs Sir A. that no accident has occurred from allowing the insane the use of the necessary instruments. The same remark applies to the Lancaster Asylum—and, in a more limited manner, to some other institutions in the country.

The great expense which will be necessary for the erection and maintenance of the projected Middlesex Asylum, has formed a ground of loud complaint. This, Sir A. thinks, is an erroneous objection. A large sum will, unquestionably, be required in the first instance, " but hereafter the parishes will be relieved from a very heavy and increasing burden." The county now pays for more than 800 pauper insane, and the number is not likely to decrease, from the modes of management and treatment pursued.

After taking a rapid survey of Lunatic Asylums in Scotland, Ireland, and several states on the Continent—from which it will be seen that, in many countries, especially in Germany, the unfortunate lunatic is still worse off than here—Sir A. throws out several valuable hints for the consideration of Parliament in the construction of the new Act. The inefficiency of the present system of licensing and inspecting mad-houses, is forcibly dwelt on. Besides the local visiters recommended by the committee, Sir A. thinks that the Secretary of State should be authorised to appoint four persons as General Inspectors for England and Wales, " who should have all the powers proposed to be given to the Local Inspectors or Visiters ; and who should be obliged to report, annually to Parliament, as is done by the Inspectors General in Ireland." He observes properly, that no establishments should be exempt from such visitations, remarks, and observations as the Inspectors may think proper to make in their annual reports. It is not necessary to give these Inspectors any controlling power over the local authorities :—" the right of visiting and examining the patients and servants will be quite sufficient for all the purposes of a general superintendence."

Sir A. has made a few observations on the nature of insanity, which are not very happily conceived, or at least expressed—though by no means erroneous in their foundation.

" A certain part or portion of the instruments of the mind are thrown from their natural position, and have, consequently, become unfit to perform their regular duties. We have two eyes, yet we only see objects single ; but let one of the eyes be pulled or pushed from its parallel movement with the other, and we immediately see the object double. In this case, the other external senses correct the defect of vision, and assure us that the order of nature, as to vision, has been interfered with, and that the impression made upon the mind is not correct; for we can feel that there is only one candle upon the table, though we see distinctly that there are two. But with regard to the intellectual faculties, whether the impressions are conveyed to the mind from external objects, or are conveyed from the mind to the external world, we have not the same means of ascertaining, that the organs or instruments that form the connexion between matter and mind have become disordered or deranged ; therefore our ideas are expressed in the form or manner in which the mind beholds them. We may suppose an individual, who, by a slight paralysis of the muscles of one eye, occurring in the dark, will see two candles, when only one is brought into the room ; and that, being unconscious of what had taken place, he would maintain that what he saw was correct, until he had explained to him the cause of such double vision, and had ascertained, by actual examination, that the fact was as so explained. So the insane person, receiving his impressions through a diseased or disordered medium, has them actually conveyed to the mind in the form in which they are expressed ; but there being no means of proving, by any other faculty, that the impression is incorrect or improper, he believes himself perfectly correct in what he expresses, and, indeed, is only expressing what he actually feels ; in the same manner as the person who saw double, without being aware of the cause, insisted that there were two objects when there was only one, because he actually saw two."

We believe with our author that, except in cases of dementia or idiotcy, from a general imperfection in the cerebral mass, we seldom find even the most furious maniac insane on more than one point. To reason with him on this point would be equally useless and ridiculous—but upon all others we should treat the madman as a human being in sound mind.

We must turn for a short time to the production of a man who humbly styles himself " a late keeper *at* a lunatic asylum." If we may judge by the language and sentiments contained in this little work, we would be inclined to suspect that the author moved in a higher circle—or, at all events, *ought* to have moved in a sphere much higher than that of a keeper *at* an asylum. Be this as it may, the anonymous author has shewn that he is intimately acquainted with the subject of which he treats—and *if* he has been only a keeper, we say that his observations are *therefore* not the less valuable.

The intelligent writer observes that lunatic asylums are springing up in every part of the country; but queries whether they are called for by the actual increase of insanity—are instituted in consequence of the inefficient systems pursued in those already established—or for "the incarceration of many persons who cannot consistently be brought under the denomination of lunatics." In support of this last accusation, for it amounts to little less, the author makes the following averment. "I have myself seen persons in lunatic asylums, for whose confinement I could not see even a colourable necessity." We can hardly doubt the truth of this statement when we reflect that a man may be incarcerated by the certificate of a chemist's apprentice just emancipated from his indentures!

"It is doubtless a matter of very great convenience to many persons, that there are places to which troublesome or eccentric relatives or friends may be packed off out of the way: and it is probably, at times, very convenient for some people to have the management of the property of others. An unfeeling and brutal husband may exasperate a sensitive and amiable woman by his neglect and cruelty, until with continued harassings, her mind becomes irritable and unsettled; she may be in the way on other accounts, and the husband may be anxious to get rid of her; he tells his own tale to a pliant physician, who calls to examine her, and the very questions he puts, implying doubts of her sanity, excite her to give irritable and incoherent replies, and she unconsciously seals her own doom; and the gloomy walls of a mad-house, the association with lunatics, and a strait waistcoat, soon finish the work which villainy began."

This may be said to be an extreme case; but our author questions "whether it is not with these extreme cases, that half our mad-houses are filled." He adduces the case of an elderly female now confined in a lunatic asylum, who, in every instance, conducts herself with the most scrupulous propriety. Her only point of weakness is, the notion that she is a lady of rank and title—and that unattended with a single circumstance which could render her obnoxious to society. It is certainly a great shame that people of this description should be immured in those places where every thing around them tends to embitter the remaining hours of their existence. It is the duty of all medical men connected with such asylums, either as attendants or as inspectors, to remonstrate against such abuses.

"Another instance I may mention, of a fine youth about twenty years of age, who is in every respect in full possession of his mental faculties; and whose only disorder appears to be deficiency of mental energy. It cannot even be called weakness of intellect, for when he is by any means roused to exertion, he not only evinces a sound judgment and discriminating powers, but is capable for a time of considerable mental labour, in studying a language for instance, or any other subject to which he may apply himself. Can any one suppose for a moment that a mad-house is a proper place for such a character as this?"

In these cases, our author does not see that any positive blame attaches to the proprietors or keepers of asylums—"they are mere passive agents." This we grant. They depend, for their existence, on the patronage of the medical profession, and it would be too much to expect of human nature, that they should run counter to their own interests, and dictate to the attesting physician or surgeon. Our author informs us that, in many instances, the form of the physician's certificate is dispensed with altogether—in other instances, the chemist or his apprentice performs the ceremony of deposition!

"It is true that commissioners are appointed, whose duty it is to inspect lunatic asylums, and to scrutinize their management; but their visits are like those of angels,

"Few and far between;"

and are generally mere visits of courtesy; for as to any useful purpose, their control is a mere dead letter, inasmuch as they have no power to inflict a penalty, or even to refuse a licence for the future, however gross may be the abuses they have detected."

The author makes many judicious observations on incipient insanity and its causes. His experience corroborates that of many others as to the effect of religious enthusiasm in deranging the mental faculties, especially among the weaker sex. He properly condemns the practice, too often pursued, of putting persons in this state into public asylums, where all chance of cure is taken away by the scenes that surround them.

"I have witnessed cases in which the most serious consequences have ensued from placing patients in an incipient state of insanity in contact with confirmed lunatics. I remember a gentleman whose disorder was an excessive state of nervous irritability; he was a tradesman in affluent circumstances, and lived in a style suitable to his means. His friends deemed it necessary that he should be removed to a lunatic asylum; and the comforts provided for him there, were far inferior to what he had been used to at home. In a very short time the change caused an accession of excitement, so that he became troublesome, and disturbed the more peaceable patients in that part of the house in which he was lodged, and he was removed into the common ward, and placed amongst lunatics of all classes. Never shall I forget the dreadful state of agitation in which he continued during the whole of one day; he refused to sit down, or to take any food, and stood shaking like an aspen leaf, his wild eyes wandering from patient to patient, as their gestures or exclamations attracted his attention."

Our author next offers some observations on the treatment of incipient insanity. Of all means, in such cases, he considers travelling, where practicable, as the best. Next to this, he recommends seclusion with a private family in the country—or in a cottage, under the direction of a judicious keeper and a medical man. The superior skill of a physician who has the experience of a lunatic asylum, is, he thinks, more than counterbalanced by the bad effects of association with other lunatics.

The brain being the organ of thought, must, of course, be the seat of insanity. But, as our direct means of acting on this organ are very limited, we must turn our attention to the general health. "It may be safely asserted that the most important thing to be attended to, is the state of the digestive organs."

"I shall now merely recapitulate the means most likely to restore a healthy tone of mind: namely, change of scene, travelling, personal comfort, a cheerful and humane attendant, pure air, plentiful exercise, and, above all, a strict attention to the state of the alimentary canal. I have no hesitation in asserting, that, had such a system been more generally adopted, hundreds who are now doomed to perpetual confinement as confirmed lunatics, would have been restored to themselves and to society."

Upon the qualifications and duties of keepers, our author makes many judicious observations, which are highly deserving of consideration. The eighth chapter, "On the Necessity of the Medical Profession paying greater Attention to the Subject of Insanity," is not beneath the notice of the medical practitioner. The author laments that, in medical education, the disorders of the mind (we mean of course its material organ) are almost totally overlooked. There should be lectures on insanity, as well as on other points of pathology. It certainly is not to be disguised, that the generality of practitioners are at a loss when they come to treat this important class of human afflictions.

"The frequent consequence of this ignorance is, that, when a medical man is called upon to attend a patient evincing symptoms of insanity, he is at a loss how to proceed; and if, after administering a few doses of medicine, and abstracting a few ounces of blood, the patient does not improve, he becomes fearful of the results of his own ignorance, and he hurries the hapless sufferer off to a lunatic asylum, frequently thereby consigning him to the care of others equally ignorant with himself, and subjecting him to the chance of a long train of evils which I have already pointed out."

We shall conclude with the following melancholy fact, which we fear is not a solitary one:

"Some time ago I was a keeper at a private lunatic asylum in the country. One day, when the medical attendant happened to be from home, a post chaise drove up to the door, and the driver delivered an order from a medical gentleman a few miles distant, to the effect, that proper persons should be sent immediately to remove a female lunatic who was to be placed under our care. It devolved upon me to attend to this; and, accompanied by a female keeper, I proceeded in the chaise to the house where the patient resided. I was shewn into the room where she lay. I found her in a state of the most violent delirium, and already under the restraint of a strait waistcoat. Her face and head were excessively hot and flushed; she was talking incoherently; one of her eyes appeared starting from its socket, and its cornea was opaque. I felt her pulse, which I found excessively quick, small, and wiry; I then made enquiry of the attendants, and found that she had occasionally, for some months past, complained of violent pains in her head, and that she was taken much in the way in which I found her, only in the afternoon of the day before; but they did not then observe the change which had taken place in her eye; she was about forty years of age, and of a gross habit of body.

"Shortly after she was taken, a medical man was sent for, who merely prescribed a blister on the nape of the neck. She, however, got worse, and it required seven or eight persons to hold her; the medical man was again sent for in the middle of the night, when he merely put a strait waistcoat upon her, and said he would direct her removal to a lunatic asylum the next day!"

She was jolted, in this state, ten or twelve miles to an asylum, and expired in a few hours after arriving there!

"Immediately upon the death of the woman, I went to inform Mr. ———, the medical gentleman who sent the case to us, and I was desired to express a wish that a *post mortem* examination should take place. He appeared no way surprised or concerned at her death, but treated it quite as a matter of course; and when I mentioned the subject of examination, he objected to it in toto. 'What good would that do?' said he. I replied, 'In order, if possible, to discover the immediate cause of her death.' 'Oh, nonsense,' was his reply,—'if any thing is found out, it will be more than ever was yet'!!"

An examination was, however, instituted, when active inflammation of the brain was discovered!

The author concludes with some observations on drunkenness and masturbation as causes of insanity. We are quite aware that the latter vice is far more destructive of human life and human reason than is imagined. Medical *writers* are reluctant in portraying its dreadful effects—but experienced practitioners every day see the terrible consequences of this solitary vice on mind and body!

"I may, perhaps, be suspected of exaggeration, when I state what proportion the number of the insane from this cause bears to the aggregate; but I have it upon the authority of a gentleman who has had some hundreds of lunatics under his care, that full three-fourths of all recent cases, in patients from eighteen to thirty years of age, were insane from the effects of masturbation! Two-fifths also of confirmed cases may be assigned to the same cause. My own experience confirms this computation, and I think the relative numbers of males and females are about equal."

The author has appended to his work an abstract of the report of the select committee appointed to inquire into the state of the pauper lunatics in the county of Middlesex, 1827. The committee of 1815 endeavoured to draw the attention of Parliament to the following abuses—which abuses are found still to continue, after a lapse of 13 years!

"'1. Keepers of the houses receiving a much greater number of persons in them than they are calculated for; and the consequent want of accommodation for the patients, which greatly retards recovery.

"'2. The insufficiency of the number of keepers in proportion to the number of persons intrusted to their care, unavoidably leading to a proportionably greater degree of restraint than the patients would otherwise require.

"'3. The union of patients who are outrageous with those who are quiet and inoffensive.

"'4. The want of medical assistance, so applied to the malady for which the persons are confined.

"'5. The detention of persons whose minds do not require confinement.

"'6. The insufficiency of the certificates on which patients are received into madhouses.

"'7. The defective visitations of private madhouses, under the provisions of the 14 G. 3. c. 49.'"

The evidence adduced before the committee of 1827, but too fully confirms the existence of the above-mentioned abuses. At the same time, we cannot help observing, that some portions of the evidence are couched in language which betrays a spirit of calumny that ought to have put the committee on their guard. We can so clearly trace such affinity between these portions of evidence and certain inflammatory harangues in the "Journal of Defamation," that no doubt remains in our minds as to the source from whence the latter were derived. These, however, we shall pass over. The following testimony of Dr. Bright will confirm what we said respecting the abuses of certificates.

"Is there any case stated in the register, where a certificate was signed several months before it was put into execution?—'William Fox, a pauper from the parish of Lambeth, appears to be neglected by his family, and he alleged moreover, that he had not seen the medical man that signed the certificate on his last admission for several months before that period.' I recollect, not very long ago, seeing a female who had been in confinement for some time, and she stated that the medical man who signed the certificate, had just looked into her room and made a bow to her, and said no more, and upon a view so hasty as that, signed the certificate; that was the information of a person who had been an inmate in a madhouse.

"Was that a physician?—It was an apothecary in Islington. There is a case in page 237 of the Report, dated April 17th, which states that a certificate had been signed by a medical practitioner by whom the patient had not been seen since August last, the certificate being dated in March.

"With regard to the person signing the certificate, have the commissioners had occasion to remonstrate?—They have; the act is so exceedingly vague, that with great reluctance they are obliged to acquiesce in the signature of persons of whom they have no testimony as to medical fitness. There was a case, of which I was lately informed, of a person, a retail chemist and druggist, calling himself an apothecary, who induced a brother of his to sign some instrument, by which property to the amount of about 3,000*l.* was disposed of, and two days after the execution of that instrument, he took this brother to a madhouse, he himself signing the certificate as a medical person."

We have been led on, by our feelings, to an extension of this article, far beyond the boundaries which we originally intended for it. We shall offer no excuse for this trespass on the common and every-day occupation of ordinary matters in medical literature and medical science. The subject is one in which all medical men are more or less interested, and on which they are very frequently called for opinion as well as advice. It is highly incumbent on them to divest themselves of prejudice, and to calmly discriminate between use and abuse. We have had opportunities lately of visiting various lunatic asylums, public and private, and we have no hesitation in declaring that, even in those which have received the greatest degree of censure, there has been such an amelioration, that much of the evil is already abated. In respect to private asylums, we could offer testimony that would be grateful to every philanthropic breast, were we permitted to mention names. One of the hardships arising from this inquiry is, the indiscriminate opprobrium which has been cast upon all private institutions, from the abuses of a few. This, however, is an evil inseparable from all public investigations of this kind, and will only be temporary. The general good will, we hope, be permanent.

To the able work of Dr. Morrison we are unable to do justice, since the letter-press contains only Outlines of Lectures. The plates are admirably executed, and express all the great features of insanity with much fidelity and spirit. We strongly recommend this work to all those concerned in the management of the insane.
